# *MC1R* Genotype and Plumage Colouration in the Zebra Finch (*Taeniopygia guttata*): Population Structure Generates Artefactual Associations

**DOI:** 10.1371/journal.pone.0086519

**Published:** 2014-01-29

**Authors:** Joseph I. Hoffman, E. Tobias Krause, Katrin Lehmann, Oliver Krüger

**Affiliations:** 1 Department of Animal Behaviour, University of Bielefeld, Bielefeld, Germany; 2 Behavioural Ecology Group, Department of Animal Sciences, Wageningen University, Wageningen, The Netherlands; Arizona State University, United States of America

## Abstract

Polymorphisms at the melanocortin-1 receptor (*MC1R*) gene have been linked to coloration in many vertebrate species. However, the potentially confounding influence of population structure has rarely been controlled for. We explored the role of the *MC1R* in a model avian system by sequencing the coding region in 162 zebra finches comprising 79 wild type and 83 white individuals from five stocks. Allelic counts differed significantly between the two plumage morphs at multiple segregating sites, but these were mostly synonymous. To provide a control, the birds were genotyped at eight microsatellites and subjected to Bayesian cluster analysis, revealing two distinct groups. We therefore crossed wild type with white individuals and backcrossed the F1s with white birds. No significant associations were detected in the resulting offspring, suggesting that our original findings were a byproduct of genome-wide divergence. Our results are consistent with a previous study that found no association between *MC1R* polymorphism and plumage coloration in leaf warblers. They also contribute towards a growing body of evidence suggesting that care should be taken to quantify, and where necessary control for, population structure in association studies.

## Introduction

Explaining the tremendous variation in plumage colouration in birds has been a prominent research focus across a wide range of fields including sexual selection [1], speciation [2], sexual dimorphism [3] and the evolution of plumage polymorphism [4], [5]. Variation in plumage pigmentation has been documented in over 300 taxonomically diverse bird species [6] and is often causally linked to melanins, either the black to brown eumelanin or the yellow to reddish-brown phaeomelanin [7]. Over the last decade, one particular component of the melanin synthesis pathway has become the focus of attention, the melanocortin-1 receptor gene (*MC1R*) which encodes a seven-pass transmembrane G protein coupled receptor [8]. High *MC1R* activity leads to increased synthesis of eumelanin, whereas low activity leads to increased synthesis of phaeomelanin. The *MC1R* has recently been established as a key gene causally explaining colour variation in many vertebrates, with single amino acid substitutions sometimes having dramatic phenotypic effects [8], [9]. The phenotypic effects of the *MC1R*, however, do not stop at colouration, but it has been proposed that this gene might also affect behavior, immune function, the nervous system and stress response [7], [10]. It is hence no wonder that the *MC1R* has been described as providing a unique window on the genetics of evolution [8]. Thus, although colour variations need not necessarily involve the *MC1R* [11], [12], this gene remains the candidate of choice for explaining the genetic basis of melanin based plumage variation in birds [8].

The zebra finch (*Taeniopygia guttata*) provides an ideal model avian system in which to explore the role of the *MC1R* in plumage colouration. Wild type zebra finches carry melanin based plumage ornaments that differ in the extent to which eumelanin and phaeomelanin are present [13]. These melanin-based ornaments are condition dependent in their expression and can be influenced by the social environment [14] and nutritional condition [15], although see also [16], [17]. Extensive variation in the plumage also occurs both naturally and in domesticated populations in the form of different colour morphs, of which over 30 have been documented [18].

**Figure 1:**
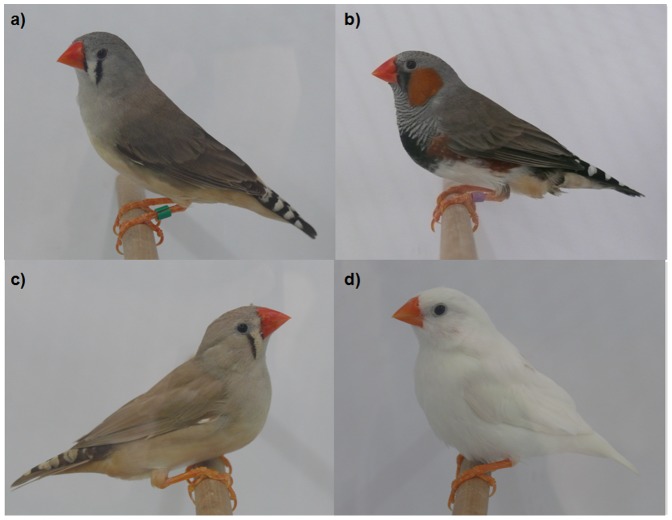
Examples of plumage coloration phenotypes found in zebra finches: (a) wild type (female); (b) black face (male); (c) fawn (female); and (d) white (female). We analysed the wild type and white plumage morphs for *MC1R* polymorphism in this study. The photographs were taken by ETK.

Wild type birds are typically greyish in colouration (Fig. 1a) with females being fully grey and males carrying additional secondary sexual ornamentation in the form of orange cheek patches, a black throat, black breast stripes and dark-reddish flanks [18]. Several of the other plumage morphs involve only minor changes to the overall plumage, an example being the black face morph, in which males carry black instead of white feathers between the tear mark and the beak and also have larger breast bands (Fig. 1b). Other plumage morphs such as silver, fawn (Fig. 1c) and white (Fig. 1d) are more extreme in that they involve changes to the entire plumage [18]. The silver and fawn morphs have greatly reduced levels of phaeomelanin and eumelanins in their feathers respectively, whereas the white morph lacks both of these pigments [19]. The white morph therefore provides the greatest contrast in terms of the level and nature of melanisation in the feathers in comparison to the wild type phenotype.

The white morph is not only a pure domesticated colour variation but it is also occasionally reported in the wild [19]. First described in 1921 [18], both sexes of this morph are fully white and the males lack all or most of the secondary sexual plumage ornaments found in the wild type morph (Fig. 1d). Its inheritance mode is described as autosomal recessive [18]. Thus, comparing the *MC1R* genotypes of white with wild type birds would appear to be a promising approach to detect polymorphisms involved in plumage colouration.

One potential problem with association studies, especially in domesticated populations, is that cryptic population structure can generate spurious associations as a byproduct of genome-wide differentiation [20]. Although this may not be a cause for concern in *MC1R* studies of unstructured populations, few studies to date have either tested for background genetic divergence or been able to account for this experimentally [8]. It is unclear how important population structure could be in studies of zebra finches, as domestic stocks exhibit some structure globally but the most profound differences are at the intercontinental level (i.e. Australia versus Europe and North America) and genetic variability is universally high [21].

To explore the role of *MC1R* in zebra finch colour polymorphism, we sequenced the near-complete *MC1R* receptor in a large sample of zebra finches with either wild type or white plumage coloration derived from five stocks. We also used microsatellite genotyping to quantify background levels of genetic differentiation and conducted a controlled breeding experiment to control for divergence among stocks.

## Materials and Methods

### Ethics statement

All procedures were carried out according to the German Laws for experimentation with animals. Blood sampling was conducted with the permission of the Landesamt für Natur, Umwelt und Verbraucherschutz Nordrhein-Westfalen (permit number 84-02.05.20.12.106) and the housing and breeding of the zebra finches was carried out with the approval of the Veterinäramt Bielefeld (permit number 530.42 1630-1) according to the German Tierschutzgesetz §11. All individuals in the breeding experiment were given coconut fibres and hay for bedding and *ad libitum* access to food. These conditions can be regarded as superior to natural conditions in which birds cannot always be sure of finding food.

### Phenotypic data

Each zebra finch (*Taeniopygia guttata*) individual was assigned to either the wild type or white plumage morph by visual inspection. The plumage morphs are described in the introduction and also depicted in Fig. 1. All of the birds were photographed using a Panasonic Lumix DML-FZ50 for future reference. Minor variation in plumage colouration frequently occurs within zebra finch stocks. Because we sampled animals at random within the stocks, some of this variability was therefore incorporated into our study. Thus, 27.8% of the wild type birds carried single feathers or small patches of white, and 32.5% of the white birds carried single feathers or small patches of grey.

The 162 F0 zebra finches analysed for colour morph were derived from five different stocks as detailed in Table 1. Three of the stocks were from Bielefeld University, herein referred to as “AUS Bielefeld”, “DOM Bielefeld” and “WHITE Bielefeld” following the convention of Forstmeier et al. [21]. The AUS Bielefeld stock originates from wild Australian birds that were bred in captivity for only about ten to fifteen generations [22] and which show genetic similarity to natural Australian zebra finch populations [21]. DOM Bielefeld are domesticated zebra finches that are more genetically similar to other European domesticated strains [21]. The usual plumage colouration of the AUS and DOM Bielefeld birds is the wild type, although small numbers of individuals with white patches have been observed in both stocks. Fawn plumage (Fig. 1c) has also been observed at low frequency in the AUS Bielefeld stock but not in the DOM Bielefeld stock. WHITE Bielefeld are domesticated zebra finches with white plumage in which the males also lack sexual plumage ornamentation. WHITE Bielefeld individuals also reveal genetic similarity to other European domestic zebra finch varieties [21]. According to Forstmeier et al. [21], WHITE Bielefeld birds are a pure strain of an autosomal recessive leucistic mutant which only produces white coloured birds and occasionally white coloured individuals with single black feathers. This is supported by the observation that we have never obtained a non-white bird in several generations of controlled breeding within the WHITE Bielefeld stock. Leucism is regarded as a total loss of pigmentation in the feathers without a loss of pigmentation at the body (i.e. the WHITE Bielefeld birds are not albinos) [23].

**Table 1:** Summary of zebra finch individuals used in the first part of this study.

Plumage Morph	Stock	Number of individuals
Wild type	AUS Bielefeld	34
	DOM Bielefeld	29
	Detmold	9
	Schloss-Holte	7
	**Total**	**79**
White	WHITE Bielefeld	74
	Detmold	7
	Schloss-Holte	2
	**Total**	**83**
**Grand total**	**–**	**162**

Two additional stocks external to Bielefeld University were also included in this study. The first of these, designated “Schloss-Holte” was a private stock of domesticated zebra finches in which the individuals originated from various commercial breeders. The “Detmold” stock similarly comprised mostly domesticated zebra finches obtained from commercial breeders, although this was augmented several years ago with individuals from the AUS Bielefeld and DOM Bielefeld stocks. Both the Schloss-Holte and the Detmold stocks included birds with wild type and white plumage colouration.

### Blood sampling, DNA extraction and *MC1R* sequencing

The brachial vein of each bird was punctured with a hydrodermic needle (Sterican, Size 20, Braun Melsungen AG) and approx. 5 µl of blood was collected using micro haematocrit tubes (Brand GmbH+Co KG, Wertheim #7493-11). Blood samples were then stored individually in phosphate buffered saline (10 mM PBS+6 mM EDTA, pH 7.4) at −20°C. Total genomic DNA was extracted using a standard phenol-chloroform protocol [24]. The full length (945 bp) *MC1R* receptor sequence (the *MC1R* does not contain any introns) was downloaded from Genbank (accession number NC_011475, chromosome *Tgu*11, 11,645,486–11,646,430 in the WUSTL v3.2.4 assembly) and 919 bp of contiguous sequence (corresponding to sites 27–945 in the sequence) was generated for each individual using two sets of primer pairs (5′-ctgcgtgagccctcgaat-3′/5′-gcgtcatgatgctgtggtag-3′ and 5′-gtcgaccgctacatcaccat-3′/5′-taccaggagcacgtcaccac-3′) designed to PCR amplify two overlapping fragments of length 439 and 533 nucleotides respectively. Each PCR was conducted in a 10 µl reaction volume containing 100 ng of template DNA, 20 mM Tris-HCl (pH 8.3), 100 mM KCl, 2 mM MgCl_2_, 0.1 mM EDTA, 0.25 mM dNTPs, 0.25 µM of each primer and 0.5 units of 5PrimeTaq polymerase (VWR). The following PCR profile was used for the first fragment: one cycle of five minutes at 94°C; 35 cycles of 30 s at 94°C, 60 s at 65°C and 60 s at 72°C; and one final cycle of seven minutes at 72°C. For the second fragment, we used one cycle of five minutes at 94°C; 30 cycles of 30 s at 94°C, 60 s at 69°C and 60 s at 72°C; and one final cycle of seven minutes at 72°C. 10 µl of the resulting PCR product was purified using shrimp alkaline phosphatase and exonuclease I (New England Biolabs) following the manufacturer's recommended protocol. All fragments were then sequenced in both directions using the Applied Biosystems BigDye® Terminator v3.1 Cycle Sequencing Kit and analysed on an ABI 3730xl capillary sequencer.

### Microsatellite genotyping

Pedigree data were not available for all of the zebra finch stocks, as the majority of individuals were bred in aviaries containing numerous individuals rather than through controlled pairings. To quantify genome-wide patterns of genetic differentiation among the colour morphs and stocks, we therefore genotyped all of the birds at eight highly polymorphic microsatellite loci (see Table 2 for details). The loci were individually fluorescently labelled and PCR amplified in two separate multiplexed reactions using a Type It Kit (Qiagen) as detailed in Table 2. The following PCR profile was used: one cycle of five minutes at 94°C; eight cycles of 30 s at 94°C, 90 s at 60°C (minus 1°C per cycle) and 60 s at 72°C; 20 cycles of 30 s at 94°C, 90 s at 56°C, 60 s at 72°C; and one final cycle of 15 minutes at 72°C. PCR products were resolved by electrophoresis on an ABI 3730xl capillary sequencer and allele sizes were scored automatically using the program GeneMarker version 1.95. To ensure high genotype quality, all traces were manually inspected within GeneMarker and any incorrect calls were adjusted accordingly. The raw microsatellite data are available in Table S1 in File S1.

**Table 2:** Polymorphism characteristics of eight microsatellite loci genotyped in 162 F0 zebra finch individuals.

Locus	EMBL accession number	Chromosome	Reference	Fluorescent label used	Multiplex	Number of alleles	Allelic size range (bp)	Ho	He
Tgu 1	EF090891	*Tgu5*	Forstmeier et al. [45]	FAM	1	13	165–218	0.723	0.848
Tgu 3	EF090892	*Tgu9*	Forstmeier et al. [45]	VIC	1	15	146–182	0.792	0.881
Tgu 4	EF090893	*Tgu6*	Forstmeier et al. [45]	FAM	2	20	87–137	0.881	0.913
Tgu 5	EF090894	*Tgu1A*	Forstmeier et al. [45]	NED	2	15	143–185	0.570	0.867
Tgu 8	EF090897	*Tgu2*	Forstmeier et al. [45]	FAM	2	16	180–228	0.841	0.909
Tgu 9	EF090898	*Tgu2*	Forstmeier et al. [45]	NED	1	13	127–164	0.796	0.882
Tgu 12	EF090901	*Tgu1*	Forstmeier et al. [45]	NED	2	15	94–120	0.804	0.880
Indigo 41	AF361047	*Tgu2*	Dawson et al. [46]	FAM	1	19	292–442	0.830	0.911

Ho, observed heterozygosity; He, expected heterozygosity. All of the loci deviated significantly from HWE at *P*<0.0001.

### Sequence analysis

Complete consensus sequences based on forward and reverse reads of two overlapping PCR fragments were generated for each of the individuals using the program ChromasPro version 1.3.4. Heterozygous sites were identified as those with two peaks of roughly equal intensity but around half the intensity of a homozygote. All sequences were then imported into BioEdit version 5.0.6 [25] and aligned to the zebra finch *MC1R* reference sequence. The strength of association between genotype and phenotype (wild type versus white plumage) was quantified based on allele counts at each variable position of the *MC1R* using Fisher's exact tests. The resulting *P*-values were then adjusted for the false discovery rate (FDR) [26] using the program Q-value [27].

### Microsatellite data analysis

Genepop [28] was used to calculate observed and expected heterozygosities and to test for deviations from Hardy-Weinberg equilibrium and for linkage disequilibrium (LD), specifying a dememorization number of 10,000, 1000 batches and 10,000 iterations per batch. We also conducted a Bayesian cluster analysis of the microsatellite genotype dataset using Structure version 2.3.3 [29]. This program uses a maximum likelihood approach to determine the most likely number of genetically distinct clusters in a sample (*K*) by subdividing the dataset in a way that maximizes Hardy-Weinberg equilibrium and linkage equilibrium within the resulting clusters. We ran five independent runs each for *K* = 1–10 using 1×10^6^ MCMC iterations after a burn-in of 1×10^5^, specifying the correlated allele frequencies model and assuming admixture. The most likely *K* was then evaluated using both the maximal average value of Ln *P*(*D*), a model-choice criterion that estimates the posterior probability of the data and Δ*K*, an *ad hoc* statistic based on the second order rate of change of the likelihood function with respect to *K* [30].

### Breeding experiment

DOM Bielefeld zebra finches with wild type plumage coloration were crossed with WHITE Bielefeld zebra finches to obtain an F1 generation. Twenty zebra finches, comprising ten DOM Bielefeld and ten WHITE Bielefeld individuals were allowed to breed in three aviaries (1 m×1 m×2 m), each equipped with five wooden nest boxes (15 cm×15 cm×15 cm), generating a total of eleven broods (See Table 3 for details). Within each aviary, each sex was of only one morph and the two sexes were of different morphs to force crossing. The sex of each individual was genetically determined prior to breeding following the protocol of Griffiths et al. [31]. To verify the parentage of F1 individuals, we genotyped all of the adults and their offspring at eight microsatellites as described above. The resulting data were then analysed within the program Colony version 2.0 [32] which uses a likelihood approach to simultaneously assign each chick a most likely mother and father and to reconstruct probable families. Default settings of the program were used, although individuals were masked in the analysis in such a way that each chick was only tested against candidate parents that were present in the appropriate aviary.

**Table 3:** Details of the first phase of the breeding experiment including the numbers of DOM Bielefeld and WHITE Bielefeld individuals of each sex that were housed in three different aviaries and the number of resulting broods.

Population	Sex	Aviary		
		1	2	3
DOM Bielefeld	Male	–	4	–
	Female	3	–	3
WHITE Bielefeld	Male	3	–	3
	Female	–	4	–
Number of F1 offspring (broods)	10 (5)	9 (4)	6 (2)	

Finally, we backcrossed the F1 offspring with WHITE Bielefeld individuals (not the parents from the F1 generation), housing each pair of individuals in a cage of dimensions 83 cm×30 cm×40 cm with an attached wooden nest box. Out of the resulting 105 offspring, 95 survived to adulthood and were classified for plumage coloration (Table 4). Around 20% of these birds were classified as being of “intermediate” plumage colouration on the basis of their having more than 25% of their total plumage assigned to both categories (white and wild type). All individuals used in the breeding experiment were blood sampled, genotyped at eight microsatellites and sequenced for the *MC1R* region as described above. The resulting data were analysed as described previously.

**Table 4:** Details of the second phase of the breeding experiment including the numbers of F1 individuals and WHITE Bielefeld individuals of each sex that were pair-wise housed in cages with the opportunity to breed.

Sex	F1 Birds	WHITE Bielefeld	Pairs	Resulting number of offspring (broods)
Male	12	12	12	51 (14)
Female	9	9	9	54 (11)
Total				105 (25)

The number of broods can exceed the number of pairs because each pair was allowed to maximally reproduce twice.

## Results

To test for an association between nucleotide variation at the *MC1R* gene and plumage coloration in zebra finches, we sequenced 79 wild type and 83 white individuals originating from five different stocks at 919 bp of the coding region that includes all of the sites known to be involved in colour variation in other vertebrate species. All individuals were also genotyped at eight highly polymorphic microsatellite loci in order to ascertain background levels of genetic differentiation among the five stocks and between the two plumage morphs. A total of 28 variable sites were identified at the *MC1R*, of which six were non-synonymous (see Table S2 in File S1 for details).

Microsatellite variability was high, with each locus carrying on average 15.8 alleles (Table 2). Significant deviations from Hardy-Weinberg equilibrium (*P*<0.0001) were observed at every locus when all of the individuals were analysed together. However, separate tests for each of seven different stock/plumage morph combinations resulted in only 11 significant values at *P*<0.05 (Table S3 in File S1), three of which remained significant after table-wide FDR correction. Moreover, these significant *P*-values were not distributed consistently across loci, indicating a probable Wahlund effect.

For the pooled microsatellite dataset, 28 pairs of loci were in significant LD (*P*<0.0001). Separate tests for each of seven different stock/plumage morph combinations resulted in 29 significant values at *P*<0.05, of which 22 remained significant after table-wide FDR correction. All but two of the latter corresponded to comparisons within the WHITE Bielefeld stock. The exceptions involved loci Tgu 9 and Indigo 41, which were in significant LD in both the AUS and DOM Bielefeld stocks, consistent with their being situated close together on chromosome *Tgu*2.

### Tests of association between *MC1R* genotype and plumage morph

The strength of association between plumage coloration and genotype was quantified at each variable position of the *MC1R* using Fisher's exact tests. Sixteen sites varied significantly between wild type and white birds, eight at *P*<0.0001, one at *P*<0.001, five at *P*<0.01 and two at *P*<0.05 (Table S2 in File S1), all of which remained significant following table-wide FDR correction [26]. However, only three of these substitutions, corresponding to positions 37, 496 and 938 in the zebra finch *MC1R* sequence, were nonsynonymous. To exclude the possibility that different mutations causing the white phenotype in different stocks obscure a simple association, we restricted the white birds used in the analysis to WHITE Bielefeld birds, which are known to represent a pure strain of an autosomal recessive leucistic mutant [21]. Similar results were obtained (data not shown).

### Background levels of divergence

**Figure 2:**
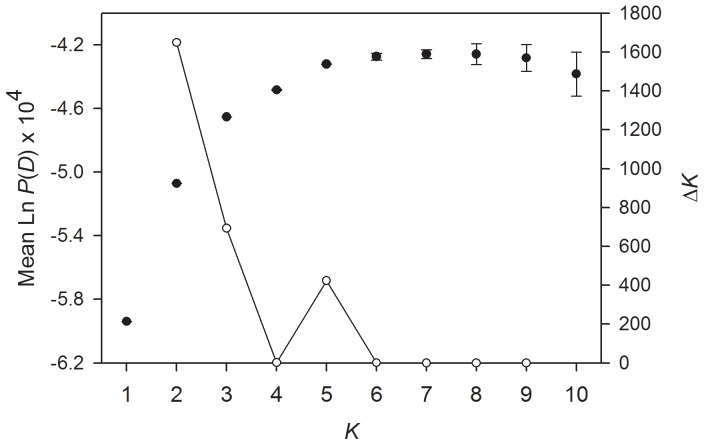
Results of the Structure analysis showing mean ± standard error Ln *P*(*D*) and ΔK values (filled and open circles, respectively) based on five replicates for each value of *K*, the hypothesized number of genetic clusters represented in the data.

**Figure 3:**
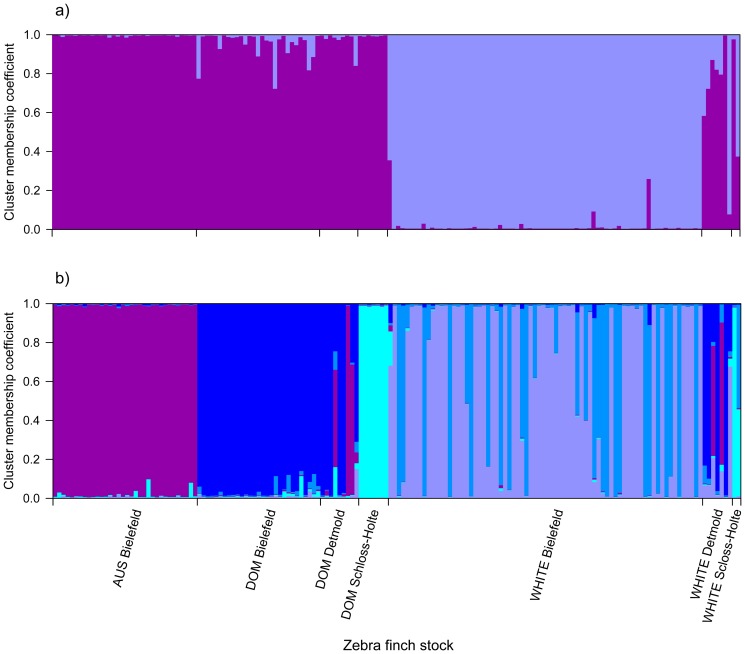
Group membership coefficients derived using the program Structure for 162 zebra finch individuals sorted by stock for (a) *K* = 2 and (b) *K* = 5. The first four stocks correspond to the wild type plumage morph and the second three stocks correspond to the white plumage morph. Each individual is represented by a vertical line partitioned into coloured segments, the lengths of which indicate the posterior probability of membership in each group.

Strong population structure has the potential to confound statistical analyses of association. Consequently, we analysed our microsatellite dataset within the program Structure [30] to determine whether any population structure could be detected without *a priori* knowledge of the stocks and plumage morphs to which individuals belonged. Five runs were conducted for each possible number of clusters (*K*), ranging from one to ten. The true number of clusters present is usually identified using the maximal value of Ln *P*(*D*), a model-choice criterion that estimates the posterior probability of the data. However, once the true value of *K* is reached, Ln *P*(*D*) often plateaus or continues to increase slightly at larger values of *K*. Consequently, we calculated Δ*K*, an *ad hoc* statistic based on the second order rate of change of the likelihood function with respect to *K* that has been shown by Evanno et al. [30] to be effective at detecting the uppermost hierarchical level of structure under most realistic scenarios. Δ*K* was maximal at *K* = 2 (Fig. 2), indicating support for two distinct genetic clusters. Membership coefficients of individuals to these inferred clusters are summarized in Fig. 3a, where each vertical bar represents a different bird and the relative proportions of the different colours indicate the probabilities of belonging to each cluster. Classifying individuals according to stock and plumage morph, a clear distinction was apparent between wild type individuals from the AUS and DOM stocks and WHITE Bielefeld individuals. Birds from the WHITE Detmold and Schloss-Holte stocks tended to be more admixed and on average appeared more similar to the AUS and DOM stocks than the WHITE stock.

Exploring population structure in greater detail, we examined the clustering pattern for *K* = 5, which corresponds to a secondary peak in Δ*K* (Fig. 2). Upon inspection, AUS Bielefeld, DOM Bielefeld, DOM Schloss-Holte and WHITE Bielefeld all emerge as clearly defined groups, although the latter is represented by two highly admixed clusters (Fig. 3b). Detmold individuals appear to be a mixture of AUS Bielefeld and DOM Bielefeld genotypes. Further increasing *K* to seven, corresponding to the maximal value of Ln *P*(*D*), did not substantially alter this pattern, with the additional clusters making negligible contributions other than to increase overall amount of admixture.

### Results of the breeding experiment

To control for the potentially confounding influence of stock structure upon tests of association between *MC1R* genotype and plumage colouration, we crossed wild type DOM Bielefeld zebra finches with WHITE Bielefeld individuals and the resulting F1 generation was backcrossed with WHITE Bielefeld individuals to generate birds with a mixed genetic background. 25 F1 offspring were obtained, of which 20 were wild type, one was white and four could not be phenotyped because they died as chicks (Fig. 4). Pairing the 21 F1s with WHITE Bielefeld birds resulted in ten pairs raising two broods, four pairs raising one brood and seven pairs failing to breed. A total of 105 chicks were obtained, of which 52 were white, 22 were wild type, 18 had intermediate plumage coloration, three were fawn and ten died before they could be phenotyped (Fig. 4). Nine segregating positions, only one of which was non-synonymous, were identified in the 92 individuals with wild type, white or intermediate plumage coloration (Table S4 in File S1). None of these sites differed significantly between either wild type and white birds, wild type and intermediate birds or white and intermediate birds (Fisher's exact tests, all *P*>0.05).

## Discussion

We tested whether *MC1R* variation explains variation in a melanin-based plumage trait in a model avian system, the zebra finch. Significant differences were observed between wild type and white birds at multiple segregating sites of the *MC1R* coding region, but these were lost after homogenising the genetic background through a controlled breeding design. Our findings are important because few if any *MC1R* association studies have been able to account for background genomic divergence via such crossing experiments.

Association tests in the F0s yielded 16 polymorphic sites that differed significantly in allele count between wild type and white birds, most of which remained so even after FDR correction. This is in contrast to the majority of previous *MC1R* association studies, which have tended to identify either a single mutation [33]–[35] or deletion [36] that is predictive of coloration. Only a handful of studies have implicated multiple polymorphisms in the *MC1R* coding region [37], [38] and for one of these it remains to be demonstrated that differences at the *MC1R* do not reflect background divergence [8]. Our results were also somewhat surprising in that only three of the substitutions significantly associated with plumage morph were non-synonymous. This suggests that the majority of sites we identified are unlikely to be of functional significance unless linked to a causative mutation elsewhere.

Bayesian cluster analysis of the microsatellite data found support for two main genetic clusters, which corresponded to birds with wild type and white plumage respectively. The only exception involved individuals with white plumage from the WHITE Detmold and WHITE Schloss-Holte stocks, which showed greater genetic similarity to wild type birds. However, this almost certainly reflects the fact that the Detmold stock has received genetic input from the AUS and DOM Bielefeld stocks. This is also apparent in the clustering result for *K* = 5 in which additional population substructure could be resolved. Notably, the majority of individuals could be confidently assigned on the basis of their cluster memberships to the AUS Bielefeld, DOM Bielefeld, Schloss-Holte or WHITE Bielefeld stocks, but birds from Detmold showed mixed contributions of AUS and DOM Bielefeld genotypes regardless of their plumage colouration.

To compensate for genetic divergence among populations, we implemented a controlled breeding experiment, crossing wild type DOM Bielefeld zebra finches with WHITE Bielefeld individuals and backcrossing the resulting F1s with WHITE Bielefeld individuals (Fig. 4). We were surprised to obtain a single bird with white plumage in the F1 generation because the white phenotype has previously been described as fully recessive [18], [19]. Similarly, 18 out of the 95 backcrosses that could be phenotyped had intermediate plumage colouration, again eliminating a straightforward dominant mode of inheritance. Three of these birds were also fawn, suggesting that the sex-linked recessive mutation [18] responsible must be segregating in the WHITE Bielefeld and possibly also the DOM Bielefeld stock despite the fawn phenotype not having been observed in either stock over at least the last five generations (E. Geissler, pers. comm.).

Intermediate plumage phenotypes in the backcrosses are suggestive of an unexpected mode of inheritance, possibly involving more than one locus. Prior to this study, however, we had no reason to believe that multiple loci could be involved, as the WHITE Bielefeld stock has previously been described as representing “a pure strain of an autosomal recessive leucistic mutant” [21]. Moreover, in several generations of controlled breeding within the WHITE Bielefeld stock, we have never obtained a non-white bird. To further explore the genetic basis of this trait, it would therefore be interesting to replicate our study using individuals of the same phenotypes but from different stocks. If the same inheritance patterns are obtained regardless of the source of the animals, this would lend support to a single locus being responsible. In contrast, if the white phenotype appears fully recessive in at least one other replicate, this would lend support to more than one mutation underlying the white phenotype in the Bielefeld WHITE stock.

Given the large number of studies reporting associations between *MC1R* genotype and coloration [8] and the fact that the white zebra finch phenotype clearly reflects a lack of melanin [13], we were surprised to find no association between *MC1R* genotype and plumage colouration. However, our data do not allow us to definitively rule out a role for *MC1R*, partly because we were unable to recover high quality sequence data for a small portion of the coding region (26/945 bp), but also because substitutions in *MC1R* regulatory regions could also be involved [8]. Nevertheless, given that we sequenced all positions known to be associated with melanin-based plumage colouration in other avian species, including all of the transmembrane domains [8], it seems unlikely that we missed a causal polymorphism within the coding region. Moreover, given that the microsatellite loci Tgu 9 and Indigo 41 are in highly significant LD (*P*<0.0001) in all of the stocks despite being approximately 5 Mb apart, an immediately adjacent causative mutation would be expected to be in strong LD with the SNPs that we assayed within the *MC1R*. Our study may therefore illustrate an important downside of the candidate gene approach, namely that of inadvertently targeting the “wrong” gene [8]. Interestingly, ours is only the second study to our knowledge not to have detected an association between sequence variation at the *MC1R* and plumage colouration [12]. Either the *MC1R* is important in the vast majority of species with melanin-based polymorphisms, or it could also be possible that studies with negative results tend to go unreported.

Although we compared the two zebra finch plumage morphs that differ the most in levels of melanin-based colouration, we also cannot rule out a role for the *MC1R* in other zebra finch plumage traits. A good candidate for further study is the black cheek morph, where the orange cheek patch in the male is instead black and females gain a black cheek patch. This particular trait is limited to a small region of plumage, it can be explained by differences in melanin deposition [13] and its expression is not sex-dependent. It may therefore have a different genetic basis to the white morph since the latter lacks multiple melanin-based ornaments, potentially implicating changes at the regulatory level.

Numerous other candidate genes could provide the basis for further study, including additional members of the melanocortin receptor family (i.e. *MC2–5R*) as well as Agouti Signalling Protein (*ASIP*), a known *MC1R* antagonist. Alternatively, given that over 40 loci control plumage coloration in chickens [39] and over 100 loci determine mouse pelage colouration [40], many other genes could potentially be involved, some of which may not necessarily be obvious candidates. For example, recent studies of white tigers [41] and gorillas [42] used whole-genome sequencing to identify single mutations within the transporter protein *SLC45A2*, which putatively affects melanogenesis by blocking transporter channel activity. We envisage such approaches being increasingly applied to unravelling the genetic basis of plumage colouration in other species. However, association studies in humans and model species suggest that high-density SNP data can also be sensitive to the confounding effects of population structure [20], [43], [44].

In conclusion, our study found no relationship between *MC1R* genotype and plumage colouration in a model avian species after having controlled for the stock of origin. This is in contrast to several other bird species in which the *MC1R* has been shown to play a pivotal role in plumage colouration, and reinforces the importance of controlling for population structure in association studies.

### Availability of supporting data

Raw microsatellite data are available in Table S1 in File S1. The *MC1R* sequence data are summarised in Tables S2 and S4 in File S1. All unique *MC1R* sequences have also been submitted to Genbank (accession numbers pending).

## Supporting Information

File S1 **Contains Tables S1–S4.** Table S1. Raw genotype data for 162 F0 zebra finches genotyped at eight polymorphic microsatellite loci. Table S2. Details of 28 segregating sites in the coding region of the *MC1R* of 161 F0 zebra finches. Note that sequence data are only available from eight of the nine wild type individuals from the Bohle private stock in Detmold, as one individual failed to generate sequence data of sufficient quality. Fisher's exact test *P*-values are given for tests of association between allele counts at each position and plumage coloration (wild type versus white). Table S3. Observed (Ho) and expected (He) microsatellite heterozygosities and *P*-values for deviation from HWE, summarised separately for each of seven different stock/plumage morph combinations. Table S4. Details of 9 segregating sites in the coding region of the *MC1R* of 92 zebra finch backcrosses. Fisher's exact test *P*-values are given for tests of association between allele counts at each position and plumage coloration (wild type versus white).
(ZIP)
