# Correction: *MC1R* Genotype and Plumage Colouration in the Zebra Finch (*Taeniopygia guttata*): Population Structure Generates Artefactual Associations

**DOI:** 10.1371/journal.pone.0096881

**Published:** 2014-04-30

**Authors:** The PLOS ONE Staff

Figure 4 is missing from the original published article. The publisher apologizes for the error. Figure 4 can be viewed here.

**Figure 4:**
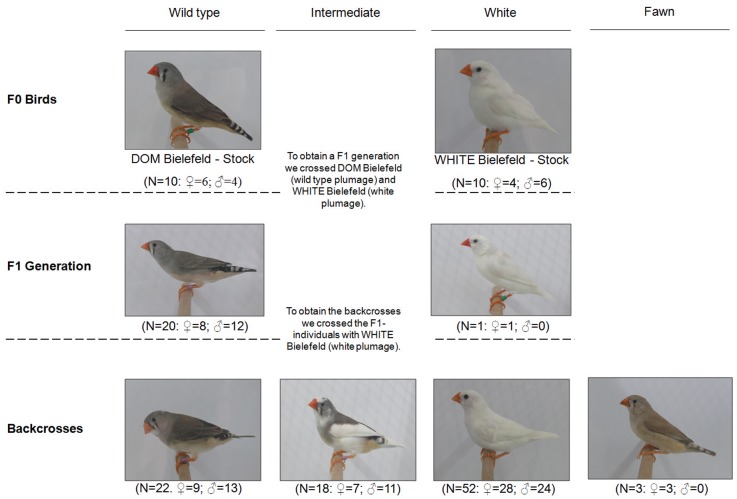
Details of the controlled breeding experiment between wild type and white zebra finches, including numbers of F1 individuals and backcrosses together with their plumage coloration phenotypes. The photographs were taken by ETK.
